# STAT3 and ERK pathways are involved in cell growth stimulation of the ST2/IL1RL1 promoter

**DOI:** 10.1002/2211-5463.12192

**Published:** 2017-01-19

**Authors:** Kenji Tago, Satoshi Ohta, Megumi Funakoshi‐Tago, Chihiro Aoki‐Ohmura, Jitsuhiro Matsugi, Shin‐ichi Tominaga, Ken Yanagisawa

**Affiliations:** ^1^ Division of Structural BiochemistryJichi Medical UniversityShimotsukeTochigiJapan; ^2^ Department of Hygienic ChemistryFaculty of PharmacyKeio UniversityMinato‐kuTokyoJapan; ^3^ Medical BiochemistryDepartment of BiochemistryJichi Medical UniversityShimotsukeTochigiJapan

**Keywords:** ERK pathway, promoter, Ras, ST2/IL1RL1, STAT3

## Abstract

The ST2 gene was originally identified as a primary responsive gene induced by stimulation with growth factors and by oncogenic stress. The ST2 gene harbors two distinct promoters – a distal promoter and a proximal promoter. In this study, we identified a novel type of serum‐responsive element in the ST2 proximal promoter using reporter gene analysis; this element includes a possible responsive element for STAT family proteins. Indeed, enforced expression of constitutively active STAT3 activated this promoter element and induced the expression of ST2 gene products. Furthermore, an oncogenic Ras (G12V) mutant also caused the expression of ST2 gene products by utilizing the proximal promoter. We also clarified that activation of the ST2 promoter by either growth stimulation or oncogenic Ras was suppressed by the inhibitors for STAT3 and ERK pathways. Our observations strongly suggest the importance of STAT family and ERK pathways for the induction of ST2 gene products by cell growth stimulation.

**Abbreviations**
ERK: extracellular signal‐regulated kinase
NP‐40: Nonidet P‐40
STAT: signal transducers and activator of transcription

ST2 (IL1RL1, also called T1) was originally identified as a primary responsive gene that was highly induced by growth stimulation and oncogenic Ras‐induced signaling in quiescent murine fibroblasts 1, 2. The ST2 gene produces two main mRNAs by alternative splicing 3; these mRNAs encode a soluble secreted protein (ST2) and a membrane bound protein (ST2L) 1, 4, 5. By structural analysis of the ST2 cDNAs, ST2 and ST2L exhibit similarity with the IL‐1 receptor (IL‐1R) suggesting that the ST2 gene products, especially membrane bound ST2L, induce similar signaling cascades to IL‐1R. After identification of IL‐33 as a specific ligand for ST2/ST2L, ST2L stimulated by IL‐33 was demonstrated to activate the transcription factor NF‐κB and the mitogen‐activated protein kinase (MAPK) family through common signaling molecules to IL‐1R 6, 7, 8, 9. On the other hand, secreted ST2 protein functions as a decoy receptor for IL‐33 and negatively regulates the signaling pathway of ST2L 10, 11. However, the functional relationship between growth stimulation and the expression of ST2 gene products is still unknown.

It was reported that the ST2 gene products are specifically expressed in type 2 helper T cells (Th2), but not in Th1, suggesting that ST2 gene products also play important roles in the immune response of Th2 12, 13, 14. Recent studies reported the expression of ST2 gene products and their physiological functions in immune/inflammation‐related cells 15, 16. Human and mouse ST2 genes have two alternative promoters followed by distinct noncoding first exons called E1a and E1b 17. In a human fibroblast cell line, ST2 gene expression was driven only by the proximal promoter. On the other hand, in the case of hematopoietic cells, both the distal and proximal promoters were utilized for the expression of ST2 and ST2L 17. These observations suggested that ST2 promoter usage depends on the cell type. In various hematopoietic cell lines, the GATA transcription factor family exhibits important roles in ST2 gene expression 18, 19. On the other hand, it has not been clarified yet how the proximal promoter is regulated, because various promoter elements have been identified in the proximal promoter of the ST2 gene 20, 21.

In the current study, we analyzed the regulatory mechanism of the expression of ST2 and identified a novel serum‐responsive element (SRE) in the proximal ST2 promoter. Furthermore, this promoter element includes a possible STAT family binding sequence, and the enforced expression of constitutively active mutant of STAT3 enabled activation of the proximal ST2 promoter.

## Materials and methods

### Cell culture and transfection

Human fibroblast TM12, murine fibroblast NIH‐3T3, and HEK293T cells were maintained in Dulbecco's modified Eagle's medium (DMEM) supplemented with 10% FBS, 2 mm glutamine, and 100 units each of penicillin and streptomycin. Transfection for the reporter gene assay was performed using the calcium phosphate method or Fugene 6 (Promega, Fitchburg, WI, USA). Murine thymoma cell line EL‐4 was cultured in RPMI1640 containing 10% FBS and 55 μm β‐mercaptoethanol. To induce differentiation into Th2‐like cells, EL‐4 cells were treated with 20 nm 12‐myristate 13‐acetate (PMA) and 1 mm dibutyryl cAMP (Bt_2_cAMP) for 24 h. UT‐7 cells were cultured in RPMI1640 containing 10% FBS and 1 ng·mL^−1^ GM‐CSF 22. Transfection was carried out by electroporation method.

### Constructions of reporter gene vectors

DNA fragments containing the distal and proximal human ST2 promoters were obtained from genomic DNA extracted from UT‐7 cells by PCR using the primer sets described below. Primer sequences were (distal promoter) 5′‐TTTGGTACCAGAGGAAAACTAGGCTGTGC‐3′ (sense) and 5′‐TTTCTCGAGCCTCATTGGGTTGTACTTGAG‐3′ (antisense), (proximal promoter) 5′‐TTTGGTACCCATTGGGTGAGTGTGAGAATG‐3′ (sense) and 5′‐TTTCTCGAGCCAAACTGAAAGACAGAGGGGATG‐3′ (antisense). These fragments were subcloned into *Kpn*I and *Xho*I sites of a promoter‐less luciferase vector (Promega). Other fragments containing various lengths of 5′‐flanking region of the ST2 gene were amplified by PCR and subcloned as described above.

### Reporter gene analysis

Cells were transfected with luciferase vectors containing various lengths of the 5′‐flanking region of ST2, and pRL‐TK containing *Renilla* luciferase to standardize the efficiency of each transfection. After 48 h, cells were harvested and lysed in passive lysis buffer (Promega). The lysates were processed for luciferase assays. Luciferase activities were determined by measuring luminescence intensity using a Lumat LB 9507 (Berthold Japan, Tokyo, Japan). Luminescence intensities derived from the reaction of firefly luciferase were normalized with that of *Renilla* luciferase.

### Electrophoretic mobility shift assay

Cells were stimulated with 10% FBS for the indicated periods. TM12 cells were harvested and lysed in 500 μL of buffer A (10 mm Hepes‐KOH [pH 7.5], 10 mm KCl, 0.1 mm EDTA, 0.1% NP‐40, 1 mm DTT, and 5 μg·mL^−1^ aprotinin) and nuclei were collected by centrifugation at 3000 ***g*** for 1 min at 4 °C. The nuclei were suspended in 100 μL of buffer C (50 mm Hepes‐KOH [pH 7.5], 420 mm KCl, 0.1 mm EDTA, 5 mm MgCl_2_, 2% [v/v] glycerol, 1 mm DTT, and 5 μg·mL^−1^ aprotinin) and mixed with gentle rotation for 30 min at 4 °C. Then, the samples were clarified by centrifugation 10 000 ***g*** for 15 min at 4 °C and the supernatant was collected and used as the nuclear extract. A quantity of 5 μg of nuclear extract was reacted with oligonucleotides derived from the proximal promoter of ST2, which was ^32^P‐labeled using T4 polynucleotide kinase (TOYOBO, Osaka, Japan) at 30 °C for 30 min. The double‐stranded oligonucleotide used as a probe derived from human ST2 promoter was as follows: 5′‐TGTCAACATCAAGAATTCTTAGTACATGAT‐3′ (region from −130 to −101 in the ST2 proximal promoter).

### Prediction of the transcription factors activating the ST2 promoter

To clarify which transcription factors regulate ST2 promoter activity, the sequence of the fragment from −130 to −101 in the proximal promoter of the human ST2 gene was analyzed using the TFBIND website (http://tfbind.hgc.jp).

### Retrovirus production and infection

Retroviruses were prepared as described previously 23. HEK293T cells were transfected with helper retrovirus plasmids together with pBabePuro and MSCV‐ires‐Puro encoding the indicated proteins. Viruses were harvested 24–60 h posttransfection, pooled, and stored on ice. Exponentially growing cells (1 × 10^5^ cells per 60‐mm‐diameter culture dish) were infected twice at 2 h intervals with 2 mL of fresh virus‐containing supernatant in complete medium containing 1.0 μg·mL^−1^ polybrene (Sigma‐Aldrich, St. Louis, MO, USA). Infected cells were collected by puromycin selection.

### Reverse transcription‐PCR

Total RNA was extracted using TRI reagent (Sigma‐Aldrich). Single‐stranded cDNA was synthesized by reverse transcription from 2 μg of total RNA using ReverTra Ace (TOYOBO). Quantitative PCR using a KAPA SYBR Fast qPCR kit (KAPA Biosystems, Wilmington, MA, USA) was performed in a LightCycler 96 (Roche Diagnostics, Indianapolis, IN, USA) with PCR cycles set at 94 °C for 10 s, 50 °C for 15 s, and 72 °C for 1 min. The nucleotide sequences of primers used for the quantitative PCR were as follows: ST2 (forward 5′‐CAAGAAGAGGAAGGTCGAAATG‐3′ and reverse 5′‐ATGTGTGAGGGACACTCCTTAC‐3′); and ST2L (forward 5′‐CAAGAAGAGGAAGGTCGAAATG‐3′ and reverse 5′‐AGCAACCTCAATCCAGAACAC‐3′). To analyze the promoter usage for ST2 gene expression, the expression of ST2 and ST2L was detected with forward primers complementary to the distal first exon (5′‐GAATAAAGATGGCTAGGACCTCTGG‐3′) or the proximal first exon (5′‐AATGAGACGAAGGAGCGCCAAGTAG‐3′), and the reverse primers were as described previously 19. PCR products were detected by staining agarose gels with ethidium bromide. For the analysis of promoter usage of the human ST2 gene, the same protocol was utilized with murine ST2, and the sequences of utilized primers were described previously 17.

### Statistical analysis of data

In the case of reporter gene analysis, we performed the experiment individually three times, and showed the data. In the graph, error bar means standard deviation (SD, *n* = 3).

## Results

### Differential usage of the distal and proximal ST2 promoters in human fibroblastic and hematopoietic cell lines

**Figure 1:**
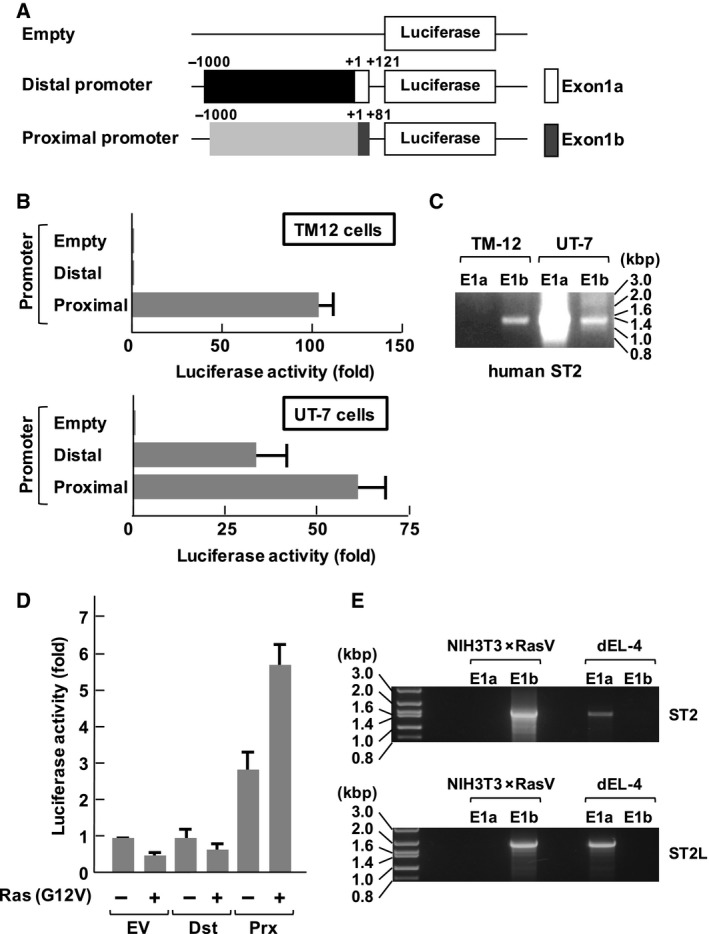
Differential usage of the distal and proximal ST2 promoters in human fibroblastic and hematopoietic cell lines. (A) The structures of luciferase vectors harboring the proximal promoters analyzed in this study are shown. An open box in the distal promoter shows the position of the distal promoter (E1a), and a gray‐colored box in proximal promoter shows the position of the proximal promoter (E1b). (B) Human fibroblasts TM12 cells and hematopoietic UT‐7 cells were transfected with the luciferase vectors shown in (A). Forty‐eight hours later, the cells were processed for reporter gene analysis. The promoter activities are shown in the graph. (C) Analysis of promoter usage of the human ST2 gene. Total RNA extracted from transformed TM‐12 and UT‐7 cells was analyzed by RT‐PCR using the primers described in the Materials and methods. The PCR products were separated on 1% agarose gels. (D) NIH‐3T3 cells were transfected with the combination of the indicated plasmids. Forty‐eight hours later, the cells were processed for reporter gene analysis. The promoter activities are shown in the graph. (E) Promoter usage of the murine ST2 gene was analyzed using total RNA extracted from transformed NIH‐3T3 × RasV and differentiated EL‐4 (dEL‐4) cells. The experiments were performed using the same method as (C). In the graph, error bar means standard deviation (SD, *n* = 3).

As reported previously, human and mouse ST2 genes have two alternative promoters, the distal and proximal promoters, followed by distinct noncoding first exons, called E1a and E1b 17. To analyze the promoter usage for the expression of ST2 gene products, we constructed separate reporter gene plasmids harboring the distal and proximal human ST2 promoters (Fig. 1A). We transfected the reporter plasmids into human fibroblasts TM12 cells and hematopoietic UT‐7 cells, respectively. Then, from performed luciferase reporter gene assays, we observed that the proximal promoter region but not the distal promoter region was activated in serum‐stimulated human fibroblast TM12 cells (Fig. 1B). On the other hand, activation of both the distal and proximal promoters was observed in UT‐7 cells. Next, we verified the promoter usage for the expression of ST2 gene products by RT‐PCR. When the distal and proximal promoters were utilized for ST2 expression, the amplification of the respective PCR products including distal first exon (E1a) and proximal first exon (E1b) were detectable. As shown in Fig. 1C, the PCR product including only E1b but not E1a was amplified from the cDNA of TM12; therefore, the expression of ST2 was driven only by the proximal promoter in TM12 cells. On the other hand, both the distal and proximal promoters were utilized for the expression of ST2 in UT‐7 cells. These results were consistent with a previous report 17. It was also reported that the expression of ST2 was induced by an oncogenic Ras mutant in NIH‐3T3 cells 2. Next, we analyzed the promoter usage for the expression of ST2 gene products in transformed NIH‐3T3 cells. As shown in Fig. 1D, an oncogenic Ras mutant (Ras (G12V)) activated the proximal promoter but not the distal promoter. Furthermore, we investigated the usage of the ST2 promoter by performing RT‐PCR with the same procedure (Fig. 1C). Unlike the case of serum‐stimulated human TM12 cells, the oncogenic Ras mutant caused the expression of not only ST2 but also ST2L in NIH‐3T3 cells (Fig. 1E). However, in both cases for ST2 and ST2L, the PCR product including E1b only but not E1a was amplified, suggesting that the oncogenic Ras mutant utilizes the proximal promoter for the expression of ST2 gene products. We also performed an analysis with total RNA extracted from a mouse thymoma cell line, EL‐4, which differentiated in response to stimulation with PMA and Bt_2_cAMP. In differentiated EL‐4 cells, the transcripts of ST2 and ST2L contained E1a, but not E1b. These results suggested that the oncogenic Ras mutant utilizes the proximal promoter for the expression of ST2 gene products, and this seems to be mediated by a similar mechanism to ST2 expression in serum‐stimulated TM12 cells. These results concur with previous observations 17, and showed that these reporter plasmids are suitable for the analysis of ST2 promoters.

### Identification of essential sequences in the ST2 proximal promoter that function in fibroblasts

**Figure 2:**
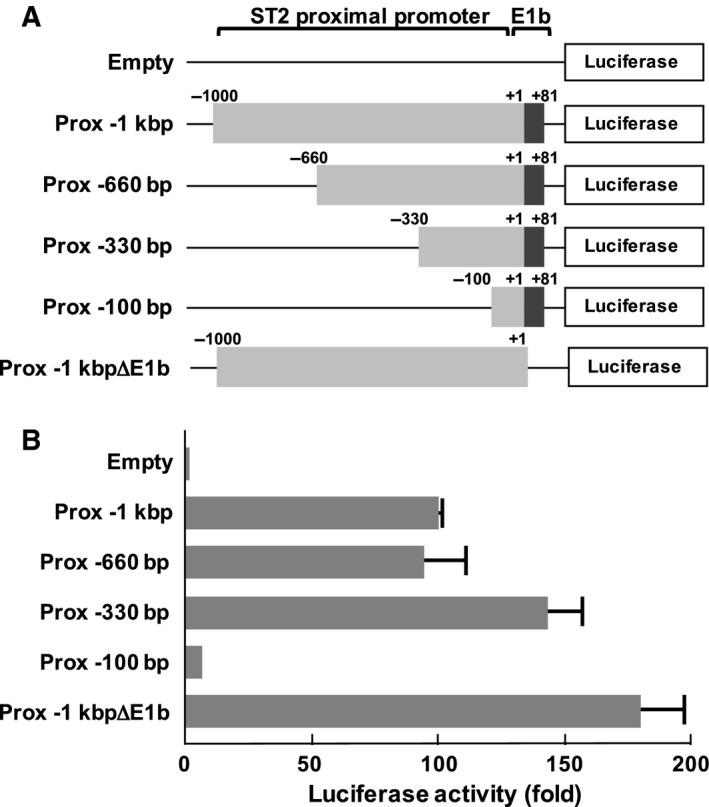
Upstream 12‐*O*‐tetradecanoylphorbol 13‐acetate‐responsive element and E‐box sequences are dispensable for activation of the proximal ST2 promoter. (A) The structures of luciferase vectors harboring proximal promoters analyzed in this study are shown. ‘Prox ‐1kbpΔE1b’ includes 1 kbp upstream from the transcriptional initiation site; however, it lacks Exon1b. The transcription initiation site is indicated as ‘+1’. The positions of promoter regions and E1b are indicated. (B) Human fibroblasts TM12 cells were transfected with the luciferase vectors shown in (A). Forty‐eight hours later, the cells were processed for reporter gene analysis. The promoter activities are shown in the graph. In the graph, error bar means standard deviation (SD, *n* = 3).

We next tried to identify the functional promoter sequences for the expression of ST2 gene products by testing the promoter activity of a series of fragments of the proximal promoter (Fig. 2A). Trüb and colleagues reported that the enhancer element [12‐*O*‐tetradecanoylphorbol 13‐acetate (TPA)‐responsive element: TRE] was located 3.6 kbp upstream of the transcription initiation site in the proximal promoter of the murine ST2 gene 20. However, a reporter gene including 1 kbp upstream from the transcriptional initiation site, which lacked the reported TRE, still exhibited high promoter activity (Fig. 2B). As result, it was clarified that the promoter fragment from −330 to −101 in the proximal ST2 promoter was enough to exhibit promoter activity. In addition, deletion of E1b (Prox ‐1kbpΔE1b) significantly enhanced the promoter activity. Unexpectedly, the fragments of the ST2 proximal promoter lacking the reported enhancer elements were still activated, suggesting that critical enhancer elements for ST2 expression seem to be located in the region closer to the transcriptional initiation site.

**Figure 3:**
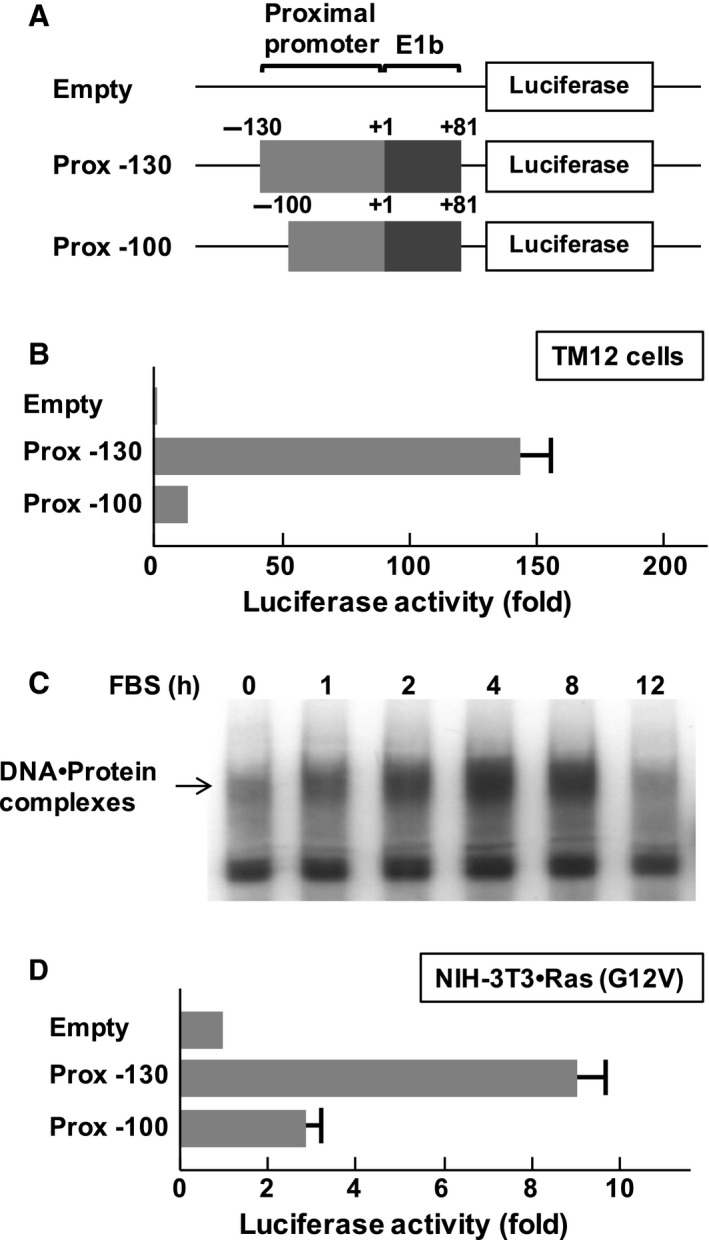
Identification of essential sequences in the ST2 proximal promoter in fibroblasts. (A) Structures of luciferase vectors analyzed in this study. Numbers indicate the position in the ST2 proximal promoter. The transcription initiation site is indicated as ‘+1’. The positions of promoter regions and E1b are indicated. (B) Human fibroblasts TM12 cells were transfected with the reporter gene plasmids. Forty‐eight hours later, the cells were harvested, and promoter activity was evaluated using luciferase assays. (C) After serum starvation, TM12 cells were stimulated with 10% fetal bovine serum for the indicated periods, and then nuclear extracts were prepared. Electrophoretic mobility shift assays (EMSAs) were performed with ^32^P‐labeled oligonucleotide probes containing the proximal promoter (−130 to −101). DNA–Protein complexes were separated on 5% nondenaturing polyacrylamide gels. (D) NIH‐3T3 cells were transfected with plasmids harboring Ras (G12V) and the indicated luciferase vectors. Forty‐eight hours later, the cells were processed for reporter gene analysis. In the graph, error bar means standard deviation (SD, *n* = 3).

Finally, we found that the region from −130 to −101 was critical for promoter activity of ST2 in TM12 fibroblasts (Fig. 3A,B). We next performed an electrophoretic mobility shift assay (EMSA) using the promoter fragment from −130 to −101 in the proximal ST2 promoter and observed the presence of DNA–protein complexes, and the formation of these complexes was dependent on serum stimulation (Fig. 3C). Furthermore, this promoter element was required for full activation of the ST2 promoter in NIH‐3T3 cells transformed with Ras (G12V) (Fig. 3D). These findings clearly showed the importance of the region from −130 to −101 in the ST2 proximal promoter for the expression of ST2 gene products in human and murine fibroblasts.

### Conserved regions in the human and mouse ST2 proximal promoters

**Figure 4:**
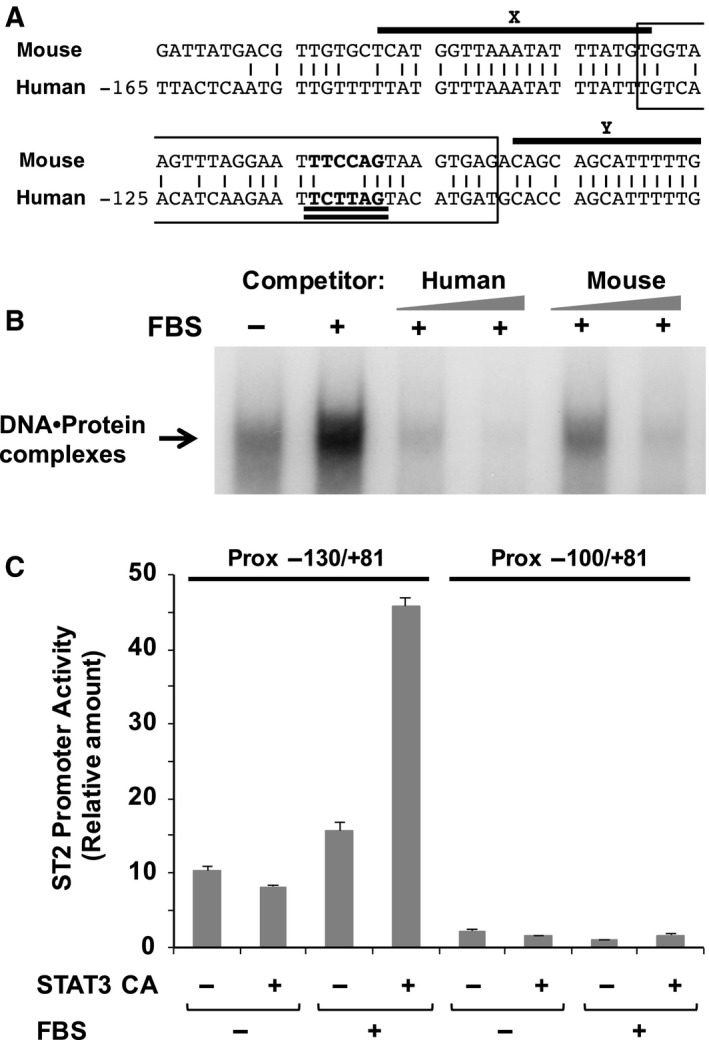
Conserved regions in the human and mouse ST2 proximal promoters. (A) The sequence similarity between the human and mouse ST2 proximal promoter is shown. The two upper‐lined sequences (X and Y) indicate the conserved regions clarified by Harr plot analysis. The boxed sequence indicates the nucleotide sequence of the probe utilized for EMSA. A possible consensus sequence for the STAT family is double underlined. (B) To show the effect of cold competitors derived from human and mouse ST2 proximal promoter on the formation of DNA–protein complexes, EMSA was performed as described in Fig. 3C and in the Materials and methods. (C) NIH‐3T3 cells were transfected with reporter gene plasmids with/without a constitutively active mutant of STAT3 (STAT3 CA). Forty‐eight hours later, the cells were harvested, and promoter activity was evaluated by performing luciferase assays. In the graph, error bar means standard deviation (SD, *n* = 3).

When comparing the nucleotide sequences between the human and murine ST2 proximal promoters, it is possible to find two close regions of high homology starting approximately 150 bp upstream and ending 50 bp upstream of the transcription initiation site of the human ST2 gene. A direct alignment of this region is displayed in Fig. 4A. The direct alignment of the promoter sequences showed the presence of the conserved regions clarified by Harr plot analysis as sequences X and Y. In the previous study, conserved region X was shown to be a critical region for the activity of the ST2 proximal promoter in growing cells, although this promoter sequence failed to respond to serum stimulation 21. We next performed EMSA to test whether a cold nucleotide probe including the similar sequence from the mouse ST2 promoter could interrupt the formation of DNA–protein complexes with the promoter fragment from −130 to −101 in the human ST2 promoter. As shown in Fig. 4B, the DNA fragment of the mouse ST2 promoter effectively interrupted the formation of DNA–protein complexes as well as the fragment of the human ST2 promoter, suggesting that a critical transcription factor for ST2 expression seems to bind to the promoter fragment from −130 to −101 in the human/mouse ST2 promoters.

### The presence of a STAT family responsive element in the ST2 proximal promoter

To clarify which transcription factors regulate ST2 promoter activity, we sought the transcription factor that is able to bind to the critical promoter sequences of both of the human and mouse ST2 genes by performing TFBIND analysis (see Materials and methods). We found a possible binding element for STAT family proteins in both the human and mouse ST2 promoter (Fig. 4A). Because STAT3 has been reported to lie downstream of the Ras signaling cascade 24, 25, we tested whether STAT3 activates the proximal promoter of the ST2 gene in murine fibroblast NIH‐3T3 cells. Strikingly, a constitutively active mutant of STAT3 stimulated the transcriptional activity from the −130/+81 fragment of the ST2 promoters but not from the −100/+81 fragment (Fig. 4C). STAT3‐mediated activation of the ST2 promoter required serum stimulation; however, STAT3 alone failed to enhance the promoter activity.

### STAT3 and ERK pathways are involved in activation of the ST2 proximal promoter

**Figure 5:**
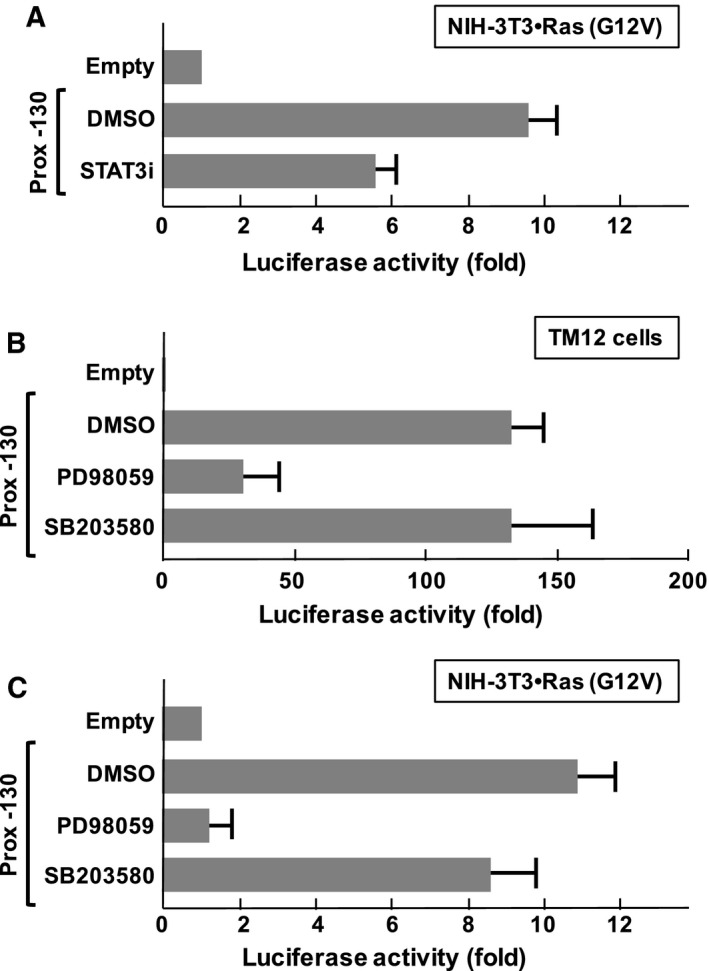
Functional involvement of STAT3 and ERK pathway in Ras (G12V)‐induced activation of ST2 promoter. (A) NIH‐3T3 cells were transfected with plasmids harboring Ras (G12V) and the indicated luciferase vectors. Then, to test the functional involvement of Ras‐related signaling molecules and STAT3, cells were treated with/without STAT3 inhibitor, 50 nm Napabucasin (STAT3i). Forty‐eight hours later, the cells were harvested, and promoter activity was evaluated by performing luciferase assays. (B) TM‐12 cells were transfected with reporter gene plasmids, and then 24 h later, cells were treated with indicated inhibitor compounds such as DMSO, 10 μm PD98059 or 10 μm SB203580. DMSO was utilized as a control. After 48 h, the cells were harvested, and promoter activity was evaluated by performing luciferase assays. (C) NIH‐3T3 cells were transfected with plasmids harboring Ras (G12V) and the indicated luciferase vectors. Then, cells were treated with the inhibitors indicated in (B). Forty‐eight hours later, the cells were harvested, and promoter activity was evaluated by performing luciferase assays. In the graph, error bar means standard deviation (SD, *n* = 3).

To gain further insight, we next investigated the involvement of STAT3 in Ras (G12V)‐induced ST2 promoter activation. Napabucasin is a potent STAT3 inhibitor 26. With napabucasin treatment, the activity of the ST2 promoter was partially suppressed in NIH‐3T3 cells transformed with Ras (G12V) (Fig. 5A). Combining with the observation in Fig. 4C, it was suggested that STAT3 harbors the ability to activate the proximal ST2 promoter and is involved in Ras‐stimulated activation of the ST2 promoter. However, the ST2 promoter was still active when STAT3 was inactivated, suggesting that additional transcription factor(s) seem to be involved in activation of the ST2 promoter. It is well established that a Ras‐induced signal activates the ERK pathway through sequential activation of c‐Raf1 and MEK1/2 27. As shown in Fig. 5B, treatment with a MEK1/2 inhibitor, PD98059, caused drastic inhibition of ST2 promoter activity in TM12 cells. On the other hand, SB203580, an inhibitor of p38 MAPK exhibited no effect. Strikingly similar results were observed in the case of NIH‐3T3 cells transformed with Ras (G12V). These observations suggested the involvement of ERK in ST2 promoter activation induced by serum stimulation and an oncogenic Ras mutant.

## Discussion

A number of studies have suggested the functional involvement of ST2 gene products and their specific ligand IL‐33 in tumorigenesis and tumor metastasis of colorectal cancer and breast cancer 28, 29, 30, 31. On the other hand, it was also reported that the IL‐33/ST2L signaling axis exhibits an antitumor function 32, 33, suggesting that the functional direction of ST2 gene products seems to depend on the environmental conditions such as the tumor cell type. Additionally, we reported recently the functional importance of ST2 for cell cycle progression provoked by serum stimulation 34, and our study suggested that ST2 seems to be involved in cell proliferation of not only tumor cells but also normal fibroblasts. These observations emphasized the importance of investigation of the mechanism by which the ST2 promoter is regulated.

Previous studies reported the important role of a 148 bp enhancer element located 3.6 kbp upstream of the transcription initiation site in the proximal promoter of the murine ST2 gene, and suggested the presence of a TRE and three E‐box sequences in this region 20, 35. These elements could be activated by the cooperative actions of AP‐1 and basic helix‐loop‐helix transcription factors 36. However, this element was not sufficient for transcription of the ST2 gene. Additionally, in our study, we found that these elements seem to be dispensable for the activation of the ST2 proximal promoter by performing reporter gene analysis (Fig. 2).

**Figure 6:**
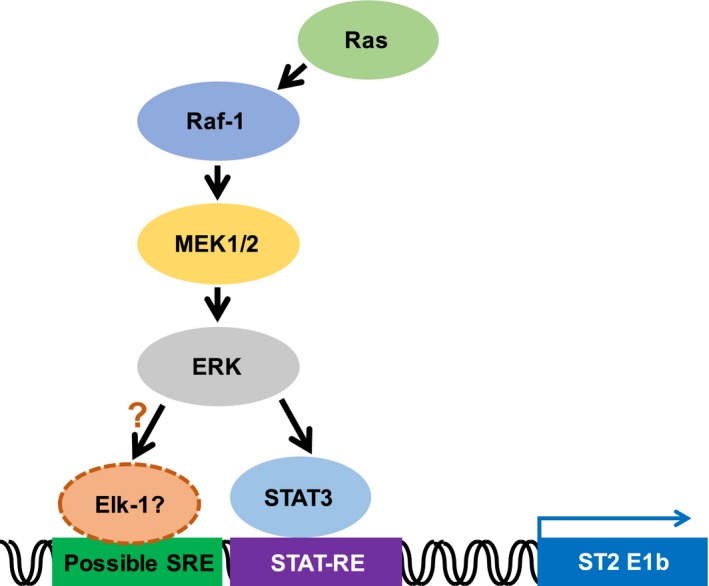
Working hypothesis how the Ras signal activates the ST2 promoter. Ras induces the expression of ST2 through the activation of the proximal promoter of ST2. The transcription factor STAT3 and ERK pathway are involved in the promoter activation.

In the current study, we identified a novel type of SRE in the proximal ST2 promoter, and this element includes a STAT family responsive element (Fig. 4A). The STAT family includes seven transcription factor proteins with high homology such as STAT1, STAT2, STAT3, STAT4, STAT5a and the closely related STAT5b, and STAT6 37. As shown in Fig. 4C, the enforced expression of STAT3 activated the proximal ST2 promoter; however, it still required serum stimulation, suggesting the requirement for additional factor(s). We found the nucleotides sequence 5′‐GGAATTTCC‐3′ in the essential region of the proximal ST2 promoter, and this is similar to the SRE. SRE is activated by the transcription factor SRF and Elk‐1 38, 39. As shown in Fig. 5B,C, ST2 promoter activation induced by serum stimulation and Ras (G12V) was effectively inhibited by treatment with PD98059, suggesting a requirement for the ERK pathway. It was reported that ERK phosphorylates and activates both STAT3 and Elk‐1 40, 41. Although we still have no direct evidence, STAT3 may cause full activation of the ST2 promoter mediated by the cooperation with Elk‐1 or SRF (Fig. 6). In addition, it is also possible that the ST2 proximal promoter could be activated by another family of STATs such as STAT5, and this possibility would need to be further investigated.

Recently, we reported that an oncogenic Ras mutant induced the expression of not only ST2 but also IL‐33 in NIH‐3T3 cells 42. Although we have no evidence at present, the expression of ST2/ST2L and IL‐33 could be regulated synchronously. Combining these reports and our current findings together, it will be important to investigate the molecular mechanism of how the expression of ST2/ST2L and IL‐33 are regulated.

## Conclusions

In the current study, we identified a novel type of SRE in the ST2 proximal promoter, which includes STAT family binding sequence. We also observed that STAT3 could activate this promoter sequence. This novel type of SRE in the ST2 proximal promoter also includes possible binding elements for SRF and Elk‐1. Using specific inhibitors, we clarified that the growth stimulation‐ and oncogenic Ras‐mediated ST2 promoter activation requires STAT3 and ERK pathway.

## Author contributions

KT and ST designed the experiments in this study. KT and CA‐O performed the experiments. MF‐T and SO prepared reagents such as retroviral vectors and plasmids. KT, JM, and KY analyzed the data. KT, ST, and KY wrote the manuscript.
